# Editorial for the Special Issue on Microfluidic Device Fabrication and Cell Manipulation

**DOI:** 10.3390/mi15010120

**Published:** 2024-01-11

**Authors:** Koji Matsuura

**Affiliations:** Department of Biosciences, Faculty of Life Science, Okayama University of Science, Okayama 700-0005, Japan; kmatsuura@ous.ac.jp; Fax: +81-86-256-9553

Microfluidic devices have been utilized for separation sciences, environmental sciences, food processing, drug delivery, bioimaging, diagnostics, and cell cultures [1,2,3,4,5,6,7,8]. In biological and biomedical applications of microfluidic devices, cells with a diameter of 1–100 µm can be manipulated by hydrodynamic forces, optical tweezers, dielectrophoresis (DEP), magnetophoresis (MP), and acoustic forces [3,4,6]. Not only controlling strategies using these forces to cells inside the microfluidic channel should be suggested in the recent manuscripts to the research community but also practical applications of the methods. The editor thinks that potential biological applications would determine the proper structures of the microfluidic devices and the suitable forces to the cells to be controlled.

Potential biological applications of microfluidic devices are point-of-care testing, biosensors, disease modeling, tissue engineering, and organ-on-a-chip [4,5,6,7]. Although laminar flow-based microfluidics are basic techniques for detection of molecules and biomolecules such as capillary electrophoresis or liquid chromatography, the microfluidic technologies for cell-based assays have become popular during the last 20 years. Especially, single cell manipulation has been emphasized to understand cellular heterogeneity [6]. The use of microfluidic devices contributes to increasing throughput and efficiency of cell analyses and mimicking physiological cell culture environments. In this research field, the device fabrication and cell manipulation using these devices are strongly related, and the researchers need to optimize their device format, including peripherical devices or instruments such as microscopes or manipulators. This Special Issue proposes examples of the integration of microfluidics and biological studies.

Figure 1 shows a word cloud prepared from the eleven abstracts published in the Special Issue, which suggests materials, the cells used in these studies, and principles for the cell manipulation. Efficiency, stability, and clinical use are important factors to apply microfluidic devices to practical biomedical applications, which are mentioned in the manuscripts. Here, the editor summarizes the materials and purposes in this Special Issue.

Target cells are blood cells, fibroblasts, endothelial cells, macrophages, hair follicles, HeLa cells, and bacteria.

Materials are polydimethylsiloxane (PDMS), polypropylene elastomer, and hydrogels (collagen, gelatin, and/or alginate).

Purpose to use the microfluidic devices are cell cultures, cellular functions, cell sorting/separation, and detection of drug resistance.

The contributions are reviewed based on the target cells and the research purposes. Song et al. used a PDMS-based 16 drug channels for single-cell-level antimicrobial susceptibility testing (Contribution 1). Hydrogel droplets or hydrogel microfluidic channels were prepared for mammalian cell manipulation in alginate microcapsules (Contribution 2), a microgel-spotting device to fabricate a multilayered gel bead culture model to mimic the early development of skin (Contribution 11) or capillary/vessel-like structures in the gelatin microstructure (Contribution 3). A free-flow measurement assay using an optical tweezer device was developed for analyzing phagocytosis of indigestible PM2.5 (Contribution 4). Feng et al. reported a 48 h cell culture on top of a microscope stage using a homemade portable cell culture device (Contribution 5). Blood cell separation (Contribution 6) and detection of tumor cells in pleural effusion (Contribution 7) were conducted using micro-structured chip devices. Red blood cell partitioning in bifurcating channels was evaluated by changing the channel dimensions (Contribution 10). Hewlin et al. reported magnetophoretic manipulation and separation of magnetic and non-magnetic particles in a simple ferro-microfluidic device (Contribution 8). A numerical simulation study aided the design of different electrostatic traveling wave electrode configurations for particle transport and biological cell manipulations (Contribution 9). Two contributions (contribution 4 and 10) were chosen as the Feature Paper and the Editor’s Choice.

The eight contributions used mammalian cells in their studies, and they were related to disease detection, tissue regeneration, or immune responses. Regarding the microfluidic device fabrication method, glass, PDMS, and cellulose paper channels have been developed for biological assay technologies [9,10]. Micro-scale structure control of hydrogels may be one of the main issues in microfluidic device fabrication and development because mammalian cells are physiologically cultured on elasticity adjusted hydrogel-like substrates to induce proper differentiation [11]. Practical applications for solving clinical problems will be proposed in future, and some research-use microfluidic devices will become a component of clinical protocols. The reported DEP and MP are also key technologies for mammalian single cell manipulation. From the emergence of lab-on-a-chip technology, biological research solutions have been provided by combinations of each technology and multiple functional physical processes [5]. 

The broad subject of this Special Issue gathers microfluidic cell manipulation research contributions that cover main research subjects in this field. Finally, the editor thanks for all the contributors to publish their manuscripts to this Special Issue.

## Figures and Tables

**Figure 1:**
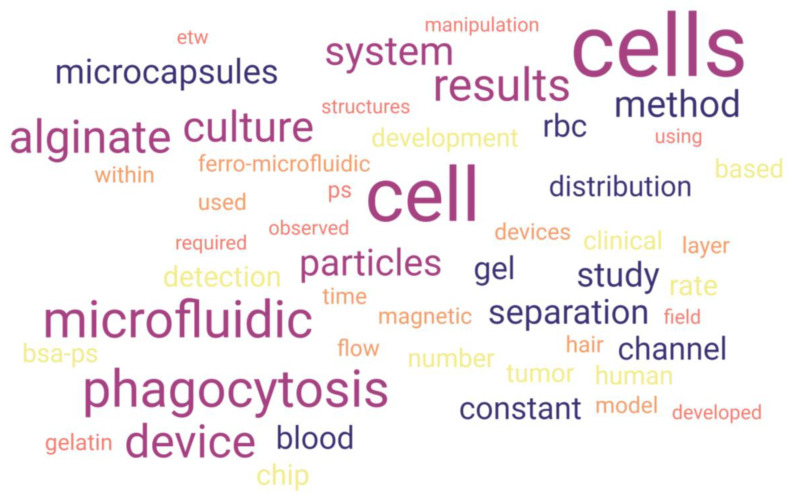
A word cloud image from the abstracts published in this Special Issue. This image was prepared using FreeWordCloudGenerator [8]. The indicated words are found more than 50 times in all the abstracts.
